# Gene expression profiling of human dermal fibroblasts exposed to bleomycin sulphate does not differentiate between radiation sensitive and control patients

**DOI:** 10.1186/1748-717X-6-42

**Published:** 2011-04-26

**Authors:** Charlotte B Westbury, Kristine Kleivi Sahlberg, Anne-Lise Borresen-Dale, Clare M Isacke, John R Yarnold

**Affiliations:** 1 Department of Radiotherapy, The Royal Marsden NHS Foundation Trust & Institute of Cancer Research, Downs Road, Sutton, Surrey, SM2 5PT, UK; 2 Breakthrough Breast Cancer Research Centre, Institute of Cancer Research, 237 Fulham Road, London SW3 6JB, UK; 3 Department of Genetics, Institute for Cancer Research, Oslo University Hospital, The Norwegian Radium Hospital, Montebello, 0310 Oslo, Norway; 4 Institute of Clinical Medicine, University of Oslo, Norway

## Abstract

**Background:**

Gene expression profiling of the transcriptional response of human dermal fibroblasts to *in vitro *radiation has shown promise as a predictive test of radiosensitivity. This study tested if treatment with the radiomimetic drug bleomycin sulphate could be used to differentiate radiation sensitive patients and controls in patients who had previously received radiotherapy for early breast cancer.

**Findings:**

Eight patients who developed marked late radiation change assessed by photographic breast appearance and 8 matched patients without any change were selected from women entered in a prospective randomised trial of breast radiotherapy fractionation. Gene expression profiling of primary skin fibroblasts exposed *in vitro *to bleomycin sulphate and mock treated fibroblast controls was performed. 973 genes were up-regulated and 923 down-reguated in bleomycin sulphate treated compared to mock treated control fibroblasts. Gene ontology analysis revealed enriched groups were cellular localisation, apoptosis, cell cycle and DNA damage response for the deregulated genes. No transcriptional differences were identified between fibroblasts from radiation sensitive cases and control patients; subgroup analysis using cases exhibiting severe radiation sensitivity or with high risk alleles present in TGF β1 also showed no difference.

**Conclusions:**

The transcriptional response of human dermal fibroblasts to bleomycin sulphate has been characterised. No differences between clinically radiation sensitive and control patients were detected using this approach.

## Introduction

Gene expression profiling of *in vitro *cellular responses of human fibroblasts and lymphocytes to radiation has demonstrated that cells undergo complex early transcriptional responses of a wide spectrum of genes from different gene ontologies [1-4]. Microarray studies have demonstrated that the transcriptional response of human cells exposed to radiation *in vitro *differs between radiation sensitive patients and controls. Therefore this approach has been explored as a predictive test of radiation sensitivity using late normal tissue effects as the endpoint of radiation sensitivity [5-7].

The spectrum of DNA damage caused by bleomycin sulphate is similar but not identical to that caused by ionising radiation, hence its definition as a radiomimetic agent [8]. The molecular and clinical responses after bleomycin sulphate and radiation are similar: both induce post-mitotic differentiation of fibroblasts inducing a senescent phenotype associated with increased collagen production [9-11], activate cascades of profibrotic chemokines and cytokines and cause skin and pulmonary fibrosis in animal models and in the clinic [12-14]. On this basis, the potential of using bleomycin sulphate rather than radiation for predictive testing is here tested in an exploratory study.

## Materials and methods

### Patients and assessment of late normal tissue injury

Patients with a history of early breast cancer treated with breast conserving surgery and radiotherapy within a clinical trial of radiotherapy fractionation were included. This patient group had prospective scoring of late normal tissue effects [15]. Using photographic scores, cases were identified as marked change in appearance (grade 3) at any assessment or a persistent moderate change (grade 2) for at least 3 consecutive years. Controls had no or minimal change to breast appearance (grade 1) and were matched to cases using defined clinical parameters [see Additional File 1].

One hundred cases with radiation change and 200 matched controls with no change were selected for translational research studies. Primary dermal fibroblasts from 26/100 best matched case control pairs were prepared from explant biopsies of buttock skin as previously described [16]. A subset of 8 case control pairs was selected for this study. Ethical approval was given by the Royal Marsden NHS Foundation Trust Ethics Committee and all patients gave written informed consent.

### Cell culture and treatment with bleomycin sulphate

Fibroblasts were seeded at passage 7-9 into T75 cm^2 ^flasks on day 0 and cultured in DMEM/10% FCS. The dose of bleomycin sulphate was previously determined by cell cycle analysis using fluorescence activated cell sorting (FACS) after treatment of fibroblasts with different doses [see Additional File 2]. On day 1, during exponential growth phase, cells were treated with 10 μg/ml bleomycin sulphate or medium alone (mock control) for 6 hours. Cells were then washed in PBS and then cultured in DMEM/10% FCS prior to RNA extraction on day 4.

### RNA extraction

Cells were washed once in PBS and lysed using the RNeasy Mini Kit (Qiagen). Briefly, cells were lysed in 600 μl extraction buffer and samples were homogenised by centrifugation in a QIAshredder (Qiagen) and stored at -80°C. For RNA isolation, 70% ethanol was added to the sample to bind the RNA to a silica membrane filter, impurities were removed by washing before finally eluting RNA in 30 μl water. RNA was concentrated in a vacuum centrifuge and analysed using an Agilent 2100 Bioanalyser (Agilent Technologies).

### RNA processing and hybridisation to Affymetrix chip

Biotinylated target RNA was prepared with minor modifications from the manufacturer's recommendations, on Affymetrix http://www.affymetrix.com/support/technical/manual/expression_manual.affx. Target RNA generated from each sample was then processed using an Affymetrix GeneChip Instrument System and hybridised to HGU133plus2 arrays. Arrays were scanned in an Affymetrix GeneChip Scanner 3000 system. Data pre-processing was carried out using Affymetrix GeneChip Operating software. The data is available on MIAMEVICE http://bioinformatics.picr.man.ac.uk/vice/PublicProjects.vice.

### Statistical analysis of Affymetrix data

Subsequent data processing was done using GCRMA package in The R project (R 2.6) http://www.r-project.org/. Data was RMA normalised and mapped to Ensembl gene IDs by Brainarray Custom CDF http://brainarray.mbni.med.umich.edu/Brainarray/Database/CustomCDF/genomic_curated_CDF.asp. Absolute values less than 50 were floored to 50. Log_2 _ratios were calculated and data was median centred. Genes for which expression values were present in less than 80% of samples were excluded. Significance Analysis of Microarrays (SAM) [17] was performed to identify genes differentially expressed between sample groups.

### Gene Ontology Analysis

To identify enriched gene ontology groups, the functional annotation tool on DAVID Bioinformatics Resources 2007 was used http://david.abcc.ncifcrf.gov/home.jsp. Enriched gene ontologies ranked highly according to statistical significance were identified (EASE score, modified Fisher exact p-value).

## Results

### Patient characteristics

Patient characteristics are shown in Table 1. Of the 8 radiation sensitive cases included, 3 cases had grade 3 scores of change in photographic breast appearance at 5 years i.e. marked radiation change. The remainder were scored as cases with moderate change (grade 2) for at least 3 successive years. All control patients had grade 1 scores i.e. no/minimal change in breast appearance.

**Table 1:** Clinical characteristics of patients incorporated into analysis.

*Cases*								*Matched control pairs*							
***Patient number***	***Age***	***Fractionation schedule***	**^*a*^*Year of follow-up***					***Patient number***	***Age***	***Fractionation schedule***	**^*a*^*Year of follow-up***				
					
		***Dose/fraction number***	***Year 1***	***Year 2***	***Year 3***	***Year 4***	***Year 5***			***Dose/fraction number***	***Year 1***	***Year 2***	***Year 3***	***Year 4***	***Year 5***

**108**	61	50 Gy/25	1	1	3	3	3	**112**	52	50 Gy/25	1	1	1	9	9

**90**	62	42.9 Gy/13	2	2	2	2	2	**75**	62	42.9 Gy/13	9	1	1	1	1

**132**	57	50 Gy/25	2	2	2	2	2	**158**	54	50 Gy/25	1	1	1	1	1

**135**	56	42.9 Gy/13	2	2	2	2	2	**144**	57	39 Gy/13	1	1	1	1	1

**137**	50	42.9 Gy/13	1	2	2	2	2	**148**	55	42.9 Gy/13	1	1	1	1	1

**138**	63	42.9 Gy/13	2	2	2	3	3	**126**	63	42.9 Gy/13	1	1	1	1	1

**115**	54	42.9 Gy/13	3	3	2	2	2	**123**	59	42.9 Gy/13	1	1	9	9	1

**98**	62	50 Gy/25	3	3	3	3	3	**106**	44	50 Gy/25	1	1	1	1	1

^*a*^Photographic scores of breast appearance, 1, no change, 2, moderate change, 3, marked change, 9, data unavailable. 10 year data was available for control patients 75, 126 and 106 - these scores were persistently 1. Matched cases and control pairs are presented in each line of the table.

### Transcriptional response of cultured fibroblasts exposed to bleomycin sulphate

To identify the transcriptional response of cells exposed to bleomycin sulphate, paired SAM of bleomycin sulphate and mock treated samples was carried out using all 16 fibroblast cultures. Of the statistically significant differentially expressed genes (false discovery rate = 0), 973 genes were up-regulated and 923 genes were down-regulated in bleomycin sulphate treated compared to mock treated fibroblasts [see Additional File 3].

For fibroblast response to bleomycin sulphate, the highly enriched gene ontologies for up-regulated genes were cellular localization and cell death (Table 2) and for down-regulated genes included regulation of progression through cell cycle, mitotic phase of cell cycle and DNA damage response and repair (Table 3).

**Table 2:** Enriched gene ontology terms for genes up-regulated in bleomycin sulphate treated fibroblasts compared to controls.

*Gene ontology term*	^a^*Number of genes*	^b^*EASE score*
Establishment of localization	187	1.01E-08

Localization	187	1.49E-08

Cellular physiological process	514	7.47E-08

Secretion	31	7.61E-08

Transport	169	3.11E-07

Protein transport	51	3.81E-07

Establishment of protein localization	52	4.27E-07

Protein localization	52	1.06E-06

Apoptosis	49	1.96E-06

Programmed cell death	49	2.17E-06

Cell death	50	2.52E-06

Death	50	3.04E-06

Secretory pathway	24	4.45E-06

Intracellular transport	50	7.70E-06

Establishment of cellular localization	50	1.07E-05

Cellular localization	50	1.33E-05

Regulation of apoptosis	34	1.57E-05

Intracellular protein transport	32	1.74E-05

Regulation of programmed cell death	34	1.77E-05

Cell organization and biogenesis	98	1.81E-05

All 16 fibroblast samples were included in the analysis. The top 20 terms for genes up-regulated after bleomycin sulphate are shown.

^*a*^The number of genes involved in the term are shown.

^*b*^The modified Fisher Exact p value (EASE score) is shown. The smaller the score, the more highly enriched the category.

**Table 3:** Enriched gene ontology terms for genes down-regulated in bleomycin sulphate treated fibroblasts compared to controls.

*Gene ontology term*	^a^*Number of genes*	^b^*EASE score*
Cell cycle	82	6.00E-16

Mitotic cell cycle	40	4.27E-15

Mitosis	33	2.77E-14

M phase of mitotic cell cycle	33	4.09E-14

M phase	36	4.38E-13

Cell division	34	7.07E-13

DNA metabolism	72	1.36E-12

Biopolymer metabolism	196	2.85E-12

Cellular physiological process	537	2.60E-11

DNA replication	33	1.52E-10

Regulation of progression through cell cycle	53	2.42E-10

Regulation of cell cycle	53	2.65E-10

Nucleobase, nucleoside, nucleotide and nucleic acid metabolism	219	1.93E-09

Spindle organization and biogenesis	11	4.02E-09

Response to DNA damage stimulus	34	1.73E-08

Response to endogenous stimulus	35	2.45E-08

Primary metabolism	395	5.41E-08

DNA-dependent DNA replication	19	5.68E-08

DNA repair	31	6.80E-08

Macromolecule metabolism	265	1.18E-07

All 16 fibroblast samples were included in the analysis. The top 20 terms for genes down-regulated after bleomycin sulphate are shown.

^*a*^The number of genes involved in the term are shown.

^*b*^The modified Fisher Exact p value (EASE score) is shown. The smaller the score, the more highly enriched the category.

### Differences between radiation sensitive patients and matched controls

SAM was used to try to identify transcriptional differences between fibroblasts isolated from radiation sensitive patients and controls. One case control pair was excluded from analysis as it was incorrectly matched for radiotherapy fractionation.

Comparisons between radiation sensitive cases and matched controls were made for mock treated fibroblast samples (i.e. not exposed to bleomycin sulphate) and for bleomycin sulphate treated fibroblast samples. No significant differentially expressed genes were identified in either comparison (data not shown).

The next approach taken was to calculate fold induction in transcript levels using gene expression ratios of log_2 _values for bleomycin sulphate treated compared to mock treated samples. Again, no significant differentially expressed genes were identified (data not shown).

Andreassen et al. reported statistically significant associations for 2 single nucleotide polymorphisms (SNPs) in TGF β1 (positions -509 and codon 10) and the risk of developing late normal tissue effects in the same patient population [18]. Of the 7 case control pairs in the current study, both high risk alleles were present in 5 cases and in 3 controls. The 5 cases with both high risk alleles and 5 matched controls were selected for further analyses. Two out of 5 of these matched controls had both high risk alleles present. SAM analysis was performed on the 5 case control pairs using fold induction values. No significant differentially expressed genes were identified (data not shown). Further analysis using 3 selected cases with marked radiation change and all 7 control patients also did not identify significant differentially expressed genes (data not shown).

## Discussion

In the current study, transcriptional profiling of dermal fibroblasts after exposure to bleomycin sulphate was carried out to determine whether differences in transcriptional response could be identified between patients with late normal tissue radiation effects and matched controls. This was a pilot study to determine if bleomycin sulphate could be used as an alternative to radiation, in this context. No differences were detected between the 2 patient groups. There are a number of possible explanations for this negative finding.

In this study, a score for late normal tissue effects was performed using photographic appearance. Cases had moderate/marked change in breast appearance and matched controls had no/minimal change. The case control selection may be a limitation of the current study. Although confounding factors such as breast size were taken into account, conventional planning techniques were used in this population leading to variations in dosimetry between cases and controls. The limitation of 5 years of follow up may have incorrectly classified radiation sensitive cases into the control group in those patients whose late normal tissue injury became manifest later. However, time to development of late normal tissue injury is a relevant parameter for judging radiation sensitivity. Other than patient 112 for whom data was missing after year 3, the available data showed no apparent injury for 4 control patients at 5 years and for 3 control patients at 10 years after radiation (Table 1).

One of the main limitations of the study was the sample size. This and the related issue of inter-sample variation may have contributed to the negative findings. An additional possible source of variation was that cells were not synchronised prior to treatment. The issue of inter-sample variation was further addressed in the microarray analysis by using fold induction values between drug and mock treated samples. The potential superiority of this approach is supported in another study of predictive testing of radiation sensitivity from the Danish cohort of breast cancer patients [19]. In this study, when cDNA array analysis of basal gene expression was compared between two patient groups, defined as radiation 'sensitive' and radiation 'resistant', only 6 genes were identified as being differentially expressed, suggesting that the difference between untreated fibroblasts from the two groups is likely to be small [6]. The authors selected 17 differentially expressed candidate genes between the two groups, identified in irradiated fibroblast samples, which were further analysed by quantitative real time polymerase chain reaction (Q-RT-PCR) [19]. The study reported that using fold induction values better differentiated radiation 'sensitive' and radiation 'resistant' patients than using either untreated samples or radiation exposed samples alone. Fold induction takes into account background levels (i.e. the transcriptional profile of untreated samples) and thereby controls for genetic variation. However in the current study, a difference between radiation sensitive cases and controls was not detected even with this approach.

Bleomycin sulphate stimulates post-mitotic differentiation of fibroblasts inducing a senescent or 'post-mitotic' phenotype associated with increased collagen production characteristic of the terminal differentiation pathway stimulated by ionising radiation [9,10,20]. In the current study, sparse cell cultures were treated for 6 hours with 10 μg/ml bleomycin sulphate on day 1 and analysis was performed at 72 hours to examine the transcriptional response of cells in the post-mitotic state. This dose of bleomycin sulphate has been previously shown to induce post-mitotic differentation in fibroblasts [9]. Under these conditions, transcriptional changes of genes related to the expression of the differentiated phenotype, considered to be relevant to late normal tissue radiation injury, may be seen. However the fibroblast response to bleomycin sulphate did not confirm enrichment of relevant gene ontology categories. For example, for up-regulated genes, the EASE score for extracellular matrix was 3.6E-02 and for response to oxidative stress was 7.3E-02.

Published data report the dose levels of bleomycin sulphate required to produce cell cycle arrest but not cell cytotoxicity. Bleomycin sulphate is known to cause both G1/S and G2/M arrest [21]. At high doses, extensive double strand DNA breaks and apoptosis occur [22]. We aimed to use a dose at which post-mitotic differentiation and cellular response pathways were induced but without causing lethality with predominant death signals. Preliminary experiments carried out in this study using FACS analysis confirmed that the dose applied resulted in predominantly G2/M arrest without significant cell death [see Additional File 2]. Using these experimental conditions, gene expression data did indeed show changes in levels of genes relevant to cell cycle control. Cell death pathway activation was also seen, and this may have contributed to the inability to differentiate radiation sensitive and control groups.

In conclusion, a difference between radiation sensitive cases and matched controls was not detected in this population of breast cancer trial patients who had prospective scoring of late normal tissue effects. This suggests any difference is likely to be small or the variation between patients too great to detect a difference. Limitations of the clinical trial design and the experimental laboratory design could have been contributory.

## Abbreviations

PBS: phosphate-buffered saline; DMEM: Dulbecco's modified eagle medium; FCS: foetal calf serum; FACS: fluorescence activated cell sorting; Q-RT-PCR: quantitative real time polymerase chain reaction; SAM: significance analysis of microarrays;

## Competing interests

The authors declare that they have no competing interests.

## Authors' contributions

CBW participated in the study design and statistical analysis, carried out cell and RNA preparation and drafted the manuscript. KKS participated in study design, performed statistical analysis and helped to draft the manuscript. ALBD particiapted in aspects of the study design and statistical analysis. JRY was responsible for conceiving the case control design and participated in the study design. CMI participated in the study design and study coordination. All authors read and approved the final manuscript.

## Supplementary Material

Additional file 1 **Details of Royal Marsden Hospital/Gloucester Oncology Centre Breast Radiotherapy Fractionation Trial (1986-1998) and scoring of late normal tissue effects**. The table includes details of the Royal Marsden Hospital/Gloucester Oncology Centre Breast Radiotherapy Fractionation Trial (1986-1998), methods of assessment of late normal tissue injury and categorisation of patients into cases with late radiation effects and controls with minimal/none. Click here for file

Additional file 2 **Cell cycle analysis of adult human dermal fibroblasts after treatment with bleomycin sulphate**. Cell cycle analysis of adult human skin fibroblasts after treatment with bleomycin sulphate was carried out. Fibroblasts were plated at a density of 2 × 10^4 ^cells in a T25 cm^2 ^flask and treated after 1 day in culture with medium alone (control), or with 10 μg/ml or 50 μg/ml bleomycin sulphate for 6 hours or 24 hours as indicated. After 4 days in culture, cells were stained with propidium iodide and analysed by fluorescence activated cell sorting (FACS); a) representative FACS profiles. b) the percentage of cells in each phase of the cell cycle determined using the Watson Pragmatic model. Both doses resulted in accumulation of fibroblasts with 4N-DNA content and therefore the lower dose (10 μg/ml) was used for the treatment of all the experimental samples. Click here for file

Additional file 3 **Genes differentially regulated between bleomycin sulphate treated and mock treated fibroblasts**. Paired SAM analysis of bleomycin sulphate treated and mock treated samples was carried out using 16 fibroblasts cultures (8 radiation sensitive cases and 8 matched controls). 973 genes were up-reulated and 923 genes were down-regulated in bleomycin sulphate treated compared to mock treated fibroblasts. Click here for file
