# Supplementary material for: Gene expression profiling of human dermal fibroblasts exposed to bleomycin sulphate does not differentiate between radiation sensitive and control patients
Source: Radiat Oncol. 2011 Apr 26;6:42. doi: 10.1186/1748-717X-6-42 (PMC3107791; doi:10.1186/1748-717X-6-42)
Supplement: Additional file 1 — Details of Royal Marsden Hospital/Gloucester Oncology Centre Breast Radiotherapy Fractionation Trial (1986-1998) and scoring of late normal tissue effects. The table includes details of the Royal Marsden Hospital/Gloucester Oncology Centre Breast Radiotherapy Fractionation Trial (1986-1998), methods of assessment of late normal tissue injury and categorisation of patients into cases with late radiation effects and controls with minimal/none. [file 1748-717X-6-42-S1.DOC]

**Additional File 1**: Details of Royal Marsden Hospital / Gloucester Oncology Centre Breast Radiotherapy Fractionation Trial (1986-1998) and scoring of late normal tissue effects.

The table includes details of the Royal Marsden Hospital / Gloucester Oncology Centre Breast Radiotherapy Fractionation Trial (1986-1998), methods of assessment of late normal tissue injury and categorisation of patients into cases with late radiation effects and controls with minimal/none.

|  | |
| --- | --- |
| Study design | Prospective randomised controlled trial of breast radiotherapy fractionation |
| Main eligibilty critera | Early breast cancer stage 1–3, maximum of one positive node and no metastasis  Local tumour excision |
| Number of randomised patients | 1410 |
| Radiotherapy fractionation schedules | 50 Gy in 2 Gy fractions, 5 weeks  39.0 Gy in 3 Gy fractions, 5 weeks  42.9 Gy in 3.3 Gy fractions, 5 weeks |
| Primary endpoint | Late change in photographic breast appearance (assessed using shrinkage relating to atrophy and distortion relating to fibrosis) |
| **Assessment of late normal tissue injury and categorisation of patients into cases and controls** | |
| Method of assessment | Post-surgical photographs of both breasts  3 point scale to assess changes in breast size and shape  None/minimal=1  Moderate=2  Marked=3 |
| Timing of assessment | Before radiotherapy, annually up to 5 years and at 10 years |
| Definition of cases | Marked change in appearance (grade 3) at any assessment or  persistent moderate change (grade 2) for at least 3 consecutive years |
| Definition of controls | No or minimal change in breast appearance (grade 1) |
| Variables used for matching cases/controls | Radiotherapy dose/fractions (50, 43, 39 Gy)  Year of follow up (1-5 years)  Width of RT field (+/-1 cm)  Thickness of lung in field (+/-0.5 cm)  Breast size (small, medium, large)  Radiotherapy boost (yes/no)  Time of scoring (1-5 years)  Hospital (Sutton, Cheltenham)  Radiotherapy field separation (+/-1 cm)  Axillary radiotherapy given (yes/no)  Tamoxifen (yes/no)  Adjuvant chemotherapy (yes/no)  Timing of chemotherapy-radiotherapy (concurrent, sequential) |
