# Supplementary material for: Gene expression profiling of human dermal fibroblasts exposed to bleomycin sulphate does not differentiate between radiation sensitive and control patients
Source: Radiat Oncol. 2011 Apr 26;6:42. doi: 10.1186/1748-717X-6-42 (PMC3107791; doi:10.1186/1748-717X-6-42)
Supplement: Additional file 2 — Cell cycle analysis of adult human dermal fibroblasts after treatment with bleomycin sulphate. Cell cycle analysis of adult human skin fibroblasts after treatment with bleomycin sulphate was carried out. Fibroblasts were plated at a density of 2 × 10^4^ cells in a T25 cm2 flask and treated after 1 day in culture with medium alone (control), or with 10 μg/ml or 50 μg/ml bleomycin sulphate for 6 hours or 24 hours as indicated. After 4 days in culture, cells were stained with propidium iodide and analysed by fluorescence activated cell sorting (FACS); a) representative FACS profiles. b) the percentage of cells in each phase of the cell cycle determined using the Watson Pragmatic model. Both doses resulted in accumulation of fibroblasts with 4N-DNA content and therefore the lower dose (10 μg/ml) was used for the treatment of all the experimental samples. [file 1748-717X-6-42-S2.PPT]

## Slide 1
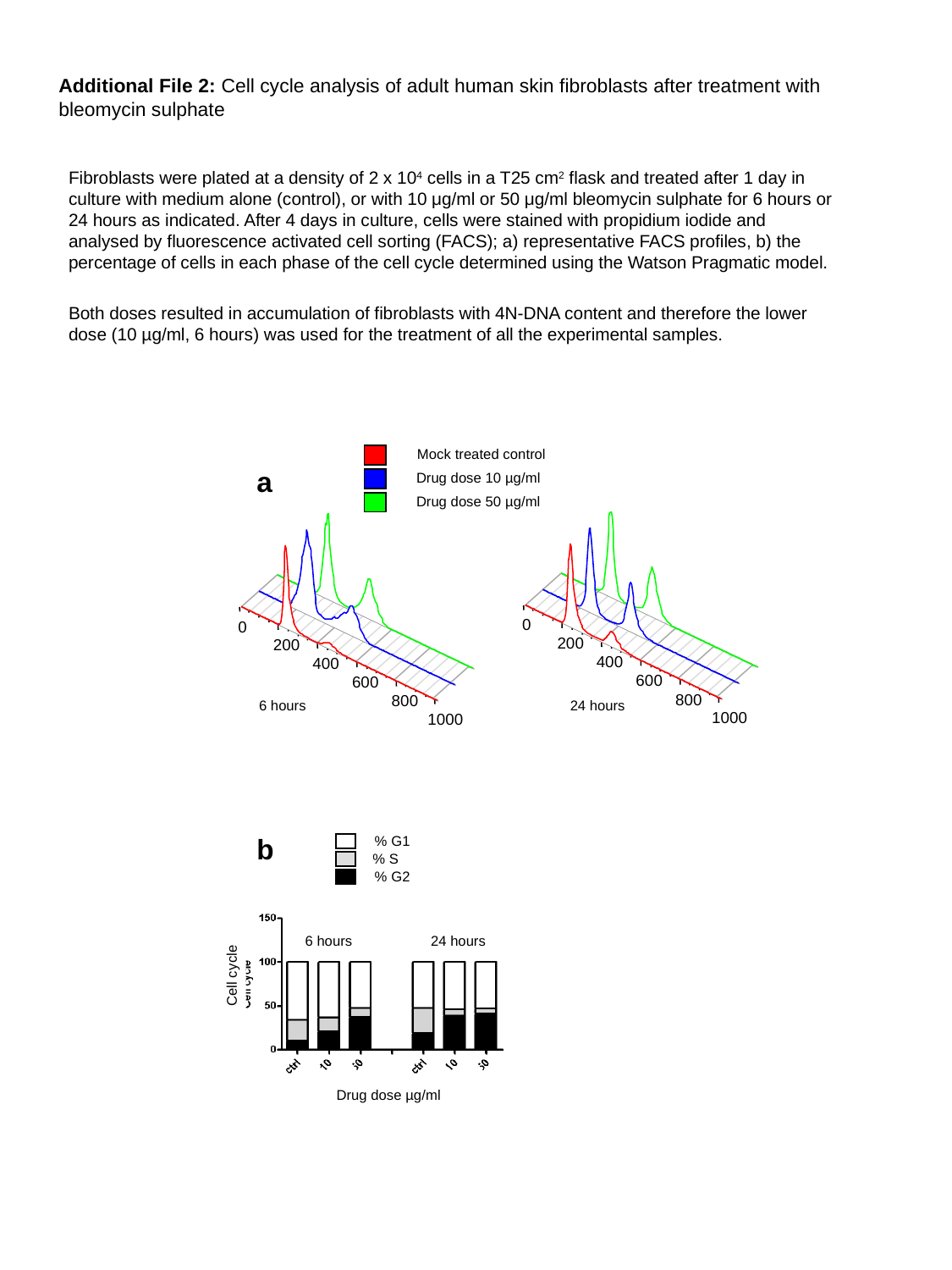

# Additional File 2: Cell cycle analysis of adult human skin fibroblasts after treatment with bleomycin sulphate
Fibroblasts were plated at a density of 2 x 10^4^ cells in a T25 cm2 flask and treated after 1 day in culture with medium alone (control), or with 10 μg/ml or 50 μg/ml bleomycin sulphate for 6 hours or 24 hours as indicated. After 4 days in culture, cells were stained with propidium iodide and analysed by fluorescence activated cell sorting (FACS); a) representative FACS profiles, b) the percentage of cells in each phase of the cell cycle determined using the Watson Pragmatic model.
Both doses resulted in accumulation of fibroblasts with 4N-DNA content and therefore the lower dose (10 µg/ml, 6 hours) was used for the treatment of all the experimental samples.
Mock treated control
Drug dose 10 µg/ml
Drug dose 50 µg/ml
a
0
200
400
600
800
1000
0
200
400
600
800
1000
% G1
% S
% G2
6 hours
24 hours
Cell cycle
Drug dose µg/ml
b
6 hours
24 hours
